# Multi‐Solvent Suppression Ultrafast 2D COSY for High‐Throughput Wine Screening

**DOI:** 10.1002/mrc.70078

**Published:** 2026-01-18

**Authors:** Pia S. Mayer, Jérémy Marchand, Marine P. M. Letertre, Jean‐Nicolas Dumez, Søren B. Engelsen, Patrick Giraudeau

**Affiliations:** ^1^ Department of Food Science University of Copenhagen Frederiksberg Denmark; ^2^ Nantes Université, CNRS CEISAM, UMR 6230 Nantes France

**Keywords:** ^1^H, 2D, COSY, high‐throughput analysis, NMR, solvent suppression, ultrafast, wine

## Abstract

Nuclear magnetic resonance (NMR) is a powerful analytical tool for wine analysis to identify and quantify a metabolite composition. However, a limiting factor of 1D ^1^H NMR spectroscopy is the overlap of signals in complex mixtures. While conventional 2D NMR methods disperse the signals over two dimensions, they are associated with long experiment times. In the case of wine, interesting metabolites are also often masked by the large water and ethanol peaks. To improve wine analysis by NMR, a method that uses the advantages of 2D NMR while suppressing solvent signals and being within the timeframe of 1D NMR is highly desirable. Interleaved ultrafast COSY (iuf‐COSY) offers a possibility for fast acquisition of a 2D spectrum and has been demonstrated as a powerful tool in metabolomics studies, as a complement to 1D NMR methods. Here, the iuf‐COSY experiment has been adapted to suppress water and ethanol signals by using a shaped pulse and a NOESY block. This approach efficiently suppresses solvent signals and gives a 2D COSY spectrum of wine in approximately 20 min. Important metabolites that originally were covered by solvent signals could be annotated, while minimal interleaving artefacts were observed. This is an efficient method to acquire a COSY spectrum of a wine sample, which can aid with the identification and discrimination of metabolites in future wine studies through additional cross peaks, while working within a high‐throughput time scale. This might be particularly interesting in the field of wine metabolomics, quality control, authenticity and fraud.

## Graphical abstract

An interleaved ultrafast COSY with multi‐solvent suppression was developed for wine analysis to overcome limitations often associated with 1D ^1^H NMR (signal overlap) and conventional 2D NMR (long acquisition times) methods. Within an acquisition time of ~20 min, it revealed hidden metabolite peaks and cross peaks for improved annotation and quantification. This method offers new possibilities for wine metabolomic studies, wine authenticity, quality control and fraud detection.

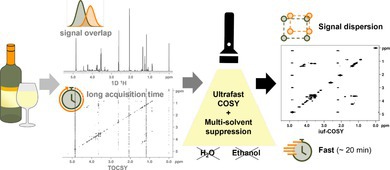

1

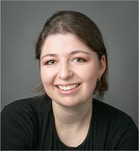

**Pia S. Mayer – MRC award at the EUROMAR 2025**

Pia is one of two winners of the MRC award at the 2025 EUROMAR conference, which recognises her work on the adaptation of the interleaved ultrafast COSY sequence to the application of wine. She completed her Bachelor‘s degree in Chemistry with Business Management from the University of Birmingham, UK, before pursuing her Master‘s degree during the COVID pandemic, at the University of Stuttgart, GER. Her thesis, supervised by Maria Buchweitz and Birgit Claasen, focused on investigating polyphenol‐protein interactions using NMR and ITC. Alongside her studies, she gained experience as a working student in the Analytical Laboratory of Mercedes‐Benz Group AG (Stuttgart, GER).

Pia is currently a final‐year PhD fellow in the Department of Food at the University of Copenhagen (DK) under the supervision of Søren B. Engelsen. Her research investigates the application of fast 2D NMR techniques combined with multidimensional chemometric tools to quantify and characterise complex mixtures in food systems. During her PhD, she visited the lab of Patrick Giraudeau in Nantes (FR), where she worked on adapting the ultrafast method. Her research explores the potential of 2D NMR methods to provide deeper insight into food composition and to address the current limitations of quantifying complex mixtures.

## 2 Introduction

Nuclear magnetic resonance (NMR) spectroscopy is a powerful analytical method for the analysis of complex mixtures. It is widely used in metabolomics, alongside mass spectrometry, for profiling metabolites with a range of applications including plant metabolism, environmental sciences and personalised medicine. Furthermore, NMR spectroscopy plays an important role in the context of food analysis and quality both in research and in industry. In particular, it can be used to verify a specific metabolite fingerprint within a sample matrix, which is interesting for metabolomic studies and important for food authenticity, food fraud, and food faults. Among others, wine is one of the product categories that is published in the monthly EU Agri‐Food Fraud Suspicions Report because of frequent adulterations [1].

NMR profiling has previously been used on wine to determine geographical origin, variety, vintages and cultivation conditions [2, 3, 4, 5]. The standard method used for untargeted analysis is a 1D ^1^H pulse sequence, due to fast data acquisition and rich information that can be extracted from the spectra [6]. However, wine, like other complex mixtures, is far from being trivial to analyse due to the variety of metabolites and the differences in concentration levels. This can lead to severely overlapped signals, limiting accurate metabolite identification and quantification. A possible solution is the addition of a second dimension to better disperse signals and improve ambiguous metabolite identification through correlation peaks. However, conventional 2D NMR methods often suffer from exceedingly long acquisition times, which contradict the principle of high‐throughput screening. For this reason, several fast 2D NMR methods have been proposed in the context of metabolite profiling [7, 8, 9, 10]. Amongst these, ultrafast 2D NMR is particularly promising for such applications since a full 2D spectrum can be recorded within a single scan [11, 12]. This can be achieved by spatially encoding the spins through the simultaneous application of gradients and frequency‐swept pulses, most commonly along the z‐axis, thereby giving them a position‐dependent evolution time. A train of bipolar gradients after the mixing time can unravel the encoded information using an echo‐planar spectroscopic imaging (EPSI) sequence. The method of choice for ultrafast sequences in the context of metabolomics has been interleaved ultrafast COSY (iuf‐COSY), which combines different hybrid strategies [13]. A first step transforms the experiment from single‐scan to multi‐scan in order to increase sensitivity by signal averaging. A second step aims to improve the observable spectral width by increasing the incremented pre‐acquisition delay before the EPSI decoding scheme.

Ultrafast and conventional COSY have previously been compared by analytical measurements and discrimination power in model mixture and metabolomic studies, showing comparable or even exceeding performance in signal‐to‐noise ratio, linearity, precision and discrimination power [8, 14, 15]. In one of the previous metabolomics studies, the combination of iuf‐COSY with an external calibration approach allowed the accurate quantification of eight target metabolites found in tomato fruit extracts, enabling quantification of metabolite concentrations down to 0.3 mM within only 5 min of acquisition [16]. Different untargeted approaches using iuf‐COSY included the profiling of lipids from serum extracts and sea sponge extracts [17, 18]. Although this method is associated with a sensitivity penalty compared to 1D ^1^H NMR, the resulting spectra are completely devoid of t_1_ noise, which is interesting for samples containing analytes with a broad span in analyte concentrations [14, 15].

In the special case of wine, the water and ethanol concentrations are several orders of magnitude higher than those of other metabolites (e.g., organic acids, sugars, alcohols, amino acids or phenolics) and give rise to three large solvent signals in the spectrum [19]. Therefore, the efficient removal of those solvent signals is necessary, and different approaches have been published in literature, such as evaporation of the solvents, freeze‐drying or instrumental multi‐solvent suppression [2, 20, 21, 22]. One advantage of NMR is the ease of sample preparation and the ability to analyse the sample ‘as is’. To preserve this benefit, an accelerated 2D method with instrumental multi‐solvent suppression would be ideal. Several suppression schemes are available, with 1D NOESY with presaturation being the most common one in metabolomics. It is simple to implement and provides efficient and repeatable suppression of faraway solvent signals [23, 24]. It has been further adapted to offer a 1D ^1^H method for the analysis of wine [2, 25].

The study extends and adapts this approach by presenting a multi‐solvent suppression interleaved ultrafast COSY method for wine analysis. It acquires a 2D spectrum in approximately 20 min and exhibits efficient water and ethanol suppression, allowing for the identification of 26 metabolites in a specific white wine sample. The newly proposed method for solvent suppression is compared to other suppression techniques that have previously been used in connection with ultrafast experiments (no solvent suppression, presaturation and WET suppression) to evaluate its potential for future high‐throughput screenings of wine samples.

## 3 Materials and Methods

### 3.1 Materials

#### 3.1.1 Chemicals

KH_2_PO_4_ (p.a. 99%, Fluka Chemie) and trimethylsilyl‐3‐propionic acid d_4_, 2,2,3,3 sodium salt ([TSP] 98% d_4_, Euroisotop) were used with D_2_O (99.9% d, Euroisotop) and ultra‐pure water (Milli‐Q, Merck) to prepare the buffer as stated in sample preparation.

#### 3.1.2 Sample Preparation

A white wine sample was prepared by mixing 700 μL of wine (Melon de Bourgogne grape from vineyard Vignoble de l'ECU, Granit, 2023 vintage, 12.5% [v/v]) with 300 μL of 1 M KH_2_PO_4_ buffer (5 mM TSP, H_2_O:D_2_O 4:1 v/v, pH = 4.3) in an Eppendorf, which had been filled with N_2_ to minimise oxidation. The sample was vortexed for 15 s, topped up with N_2_ and stored in the fridge until use. On the day of acquisition, 200 μL was transferred into a 3 mm NMR tube.

#### 3.1.3 Spectrometer

Data acquisition was performed on an Avance III HD 16.4 T spectrometer (Bruker Biospin) with an inverse ^1^H/^13^C/^15^N/^2^H (^2^H lock) cryogenically cooled probe equipped with a Z gradient coil, operating at a ^1^H Larmor frequency of 700.28 MHz and running under Topspin 3.2. All NMR experiments were conducted at 298 K.

### 3.2 Methods

In the following paragraph, the workflow of the iuf‐COSY is described, starting from the 1D optimisation processes, followed by the 2D experimental parameters and finally the processing information.

#### 3.2.1 1D ^1^H Experiments for Suppression Parameter Optimisation

##### 3.2.1.1 Presaturation Experiment

A standard presaturation experiment was used to optimise water suppression for the carrier frequency and presaturation power, as well as the 90° ^1^H pulse. The recovery delay was set to 5 s, and the acquisition time to 3.9 s with a spectral width of 12 ppm. Two scans and two dummy scans were used.

##### 3.2.1.2 Multi‐Solvent Presaturation Experiment

To optimise the multi‐solvent suppression, integral regions were defined on the resulting water presaturation spectrum. These regions were chosen to be in the middle of the ethanol triplet and quartet (7 Hz dispersion each) and on the residual water peak (20 Hz dispersion) to generate a shaped pulse using the ‘selective 1D’ set‐up tool for presaturation within Topspin. The generated multiple‐band selective shaped pulse was tested in a multi‐solvent presaturation pulse sequence that uses the shaped pulse during the relaxation delay, a NOESY block and additional spoil gradients (see Figure S1). The power of the shaped pulse was optimised for efficient suppression without baseline distortion. Acquisition parameters were as follows: Duration of the suppression shaped pulse was set to 100 ms and repeated 50 times to have a total of 5 s of relaxation delay with an acquisition time of 3 s and a spectral width of 12 ppm. Four dummy scans and eight scans were recorded with a mixing time of 10 ms between the second and the third 90° pulse. Two additional 1 ms defocusing gradients of +31.3 G/cm (G_1_) and −6.2 G/cm (G_2_) (smooth‐square shape) were placed after the shape pulse and after the mixing time of the NOESY block.

##### 3.2.1.3 WET Experiment

The pulse sequence WET was used to optimise water and ethanol suppression. It was performed with four selective Seduce pulses with a duration of 15 ms each. The power of these pulses was manually tuned to optimise the resulting solvent suppression. Otherwise, the relaxation delay was 5 s, the acquisition time was 3 s and the spectral width was 12 ppm. For optimisation purposes, only one scan was acquired.

#### 3.2.2 Set‐Up of Ultrafast COSY Experiment

Previous work has led to the current version of the interleaved ultrafast COSY (iuf‐COSY), including interleaving and multi‐scan methods [13]. A detailed implementation protocol is available online [26].

**FIGURE 1:**
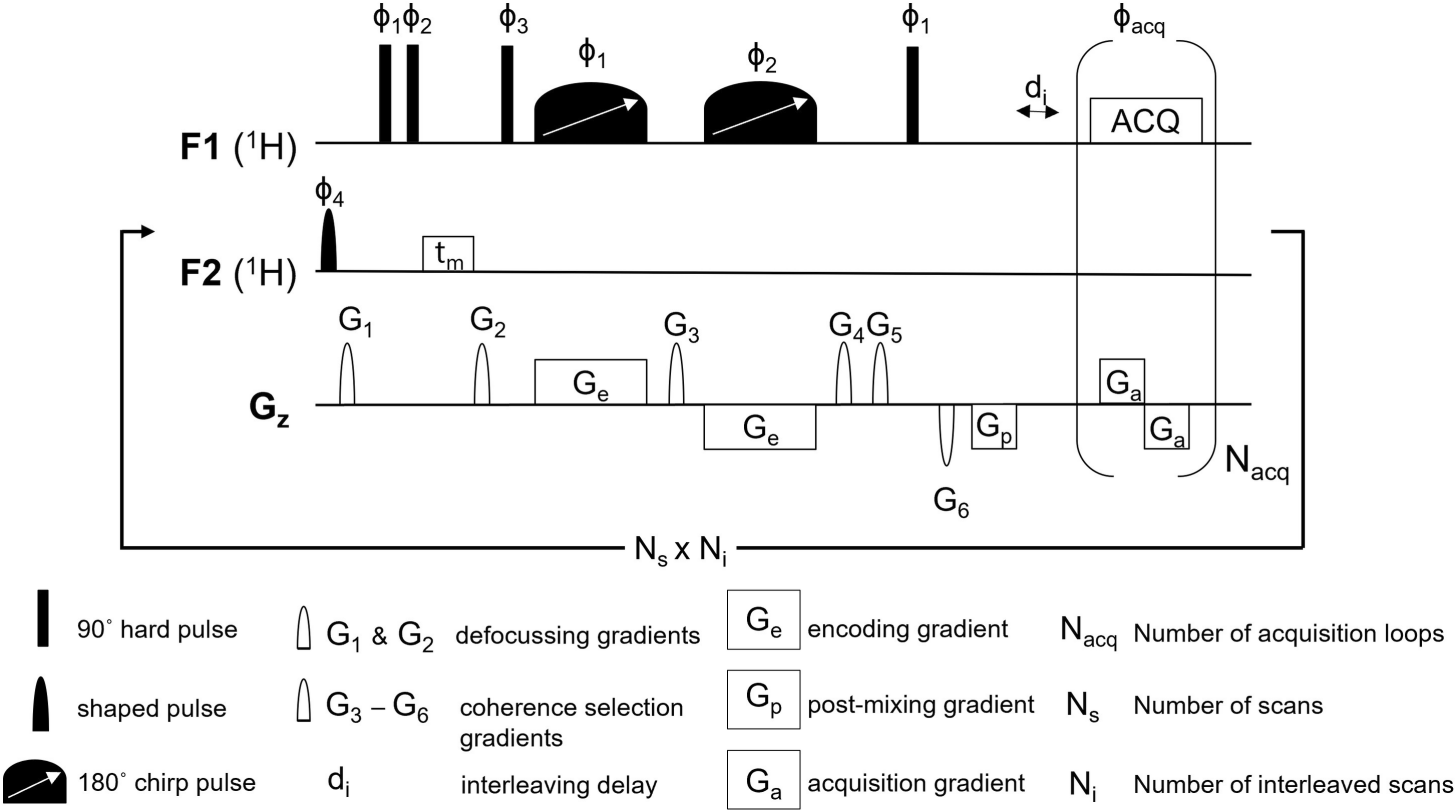
Pulse sequence of interleaved ultrafast COSY with multi‐solvent suppression (iuf‐COSY‐msup). To the general method a solvent suppression with a shaped pulse, two defocusing gradients (G_1_ and G_2_) and NOESY block (with t_m_) were added for efficient water and ethanol signal suppression. The iuf‐COSY‐msup consists of two opposing gradients (G_e_) with smoothed chirp pulses for encoding, a post‐mixing gradient (G_p_) with additional coherence selection gradients (G_3_–G_6_). An interleaving delay (d_i_) for interleaved scans (N_i_) is also used before the start of the acquisition block which features bipolar gradients (G_a_) that are repeated N_acq_ times. Phase cycling was as follow ϕ_1_ = x, −x; ϕ_2_ = x, x, x, x, x, x, x, x, −x, −x, −x, −x, −x, −x, −x, −x; ϕ_3_ = x, x, −x, −x, y, y, −y, −y; ϕ_4_ = x; ϕ_acq_ = x, −x, −x, x, y, −y, −y, y, −x, x, x, −x, −y, y, y, −y. Gradients were applied as follows: G_1_ = 31.3 G/cm for 1 ms; G_2_ = 15.6 G/cm for 1 ms; G_3_ and G_4_ = ± 50.1 G/cm for 800 μs; G_5_ and G_6_ = ±50.1 G/cm for 1 ms; G_e_ = ±3.6 G/cm for 15 ms; G_a_ = ±50.1 G/cm for 784 μs; G_p_ = −28.1 G/cm for 784 μs.

All iuf‐COSY methods share the same core (Figures 1, S2 and S3), differing only in their solvent suppression techniques. The parameters in this paragraph were identical across all iuf‐COSY experiments; otherwise, any additions or modifications are specified in the following suppression section. Ninety‐degree pulses were calibrated as mentioned before. Both encoding chirp pulses have a duration of 15 ms and a sweep range of 28 kHz and encoding gradients (G_e_) of ±3.6 G/cm, while for the acquisition (G_a_) ± 50.1 G/cm (smooth‐square shape) gradient amplitudes were applied for 748 μs. Coherence selection gradients G_3_ and G_4_ were +50.1 G/cm for 800 μs (sine shape), while coherence selection gradients G_5_ and G_6_ were ± 50.1 G/cm for 1 ms (sine shape). An acquisition time of 98 ms with 64 incremented acquisition loops (N_acq_), 8 interleaved scans (N_i_) and 32 scans (N_s_), along with 4 dummy scans and a recovery delay of 5 s, resulted in an experimental time of 23 min per spectrum. The recovery delay of 5 s was chosen as it is the minimum recommended rest time for the gradient coil between two successive ultrafast scans. All experiments were performed with five replicates.

##### 3.2.2.1 Water and No Solvent Suppression

Both spectra were recorded using the same pulse sequence based on a continuous wave presaturation pulse in F2 (^1^H) (see Figure S2). Optimised parameters from the presaturation 1D ^1^H experiment were taken for water suppression, while the power was set to 0 W for the experiments with no solvent suppression. Post‐mixing gradient (G_p_) for the iuf‐COSY with presaturation (iuf‐COSY‐pr) was set to −21.8 G/cm for 748 μs.

##### 3.2.2.2 Multi‐Solvent Suppression

This pulse sequence of iuf‐COSY with multi‐solvent suppression (iuf‐COSY‐msup) is shown in Figure 1. It consists of a shaped pulse, followed by a defocusing gradient (G_1_), two 90° pulses followed by mixing time (t_m_ = 10 ms) and a second defocusing gradient (G_2_) before the spatial encoding. Parameters and shapes were taken from optimised 1D ^1^H experiments, except for additional defocusing gradients, which were set to 31.1 G/cm (G_1_) and 15.6 G/cm (G_2_) for 1 ms (smooth‐square shape), and the post‐mixing gradient (G_p_) was set to −21.8 G/cm for 748 μs.

##### 3.2.2.3 WET Suppression

For this method, the WET block with the four composite pulses was added as a suppression technique to the iuf‐COSY (iuf‐COSY‐WET) (see Figure S3). The parameters were taken from the optimised 1D experiment. The post‐mixing gradient (G_p_) was −28.0 G/cm for 748 μs.

#### 3.2.3 Processing

##### 3.2.3.1 1D ^1^H Experiments

Processing was performed on Topspin 3.2 with a decaying exponential window function, a line‐broadening of 0.3 Hz and zero‐filling to 131,072 data points (SI 128k). The phase and baseline were automatically corrected within Topspin and manually adapted if necessary.

##### 3.2.3.2 iuf‐COSY

A custom Python processing script, running within Topspin, was used for processing all iuf‐COSY spectra. The code file is available online and was run on Topspin 3.2 and 4.3 [26]. The iuf‐COSY data were processed by an inverse Fourier transformation along the ultrafast dimensions, followed by a Gaussian apodisation in the spatial domain and a Fourier transform, while for the conventional dimension, a sine window function was applied before Fourier transformation [27]. Data were zero‐filled to 512 × 1024 data points. The chemical shift for each spectrum was manually calibrated with a custom Python script, using the TSP (0.00/0.00 ppm) and tartaric acid peak (4.47/4.47 ppm) [26].

#### 3.2.4 Metabolite Annotation

Annotation of metabolites was performed using the 1D ^1^H spectra, supplemented with additional structural information from the iuf‐COSY and a 2D TOCSY spectrum, which applied NUS and multi‐solvent suppression (Figure S4). Literature and spectral database comparison were performed, resulting in metabolite annotation of Levels 2 (25 metabolites) and 3 (1 metabolite) based on the definition of the Metabolomics Standards Initiative [28].

#### 3.2.5 Signal‐to‐Noise Ratio Calculations

To calculate the signal‐to‐noise ratio (SNR), data were transferred to Matlab (Mathworks, Version 2023a). Signals of cross peaks were preferred whenever possible, since they appear in less crowded regions and show less overlap. However, in spectra without solvent suppression, only diagonal peaks were observed, except for cross peaks from lactate. To facilitate comparison of several signals across all methods, four diagonal peaks (TSP, succinate, lactate and tartrate) and one cross peak (lactate) were selected. Additional cross peaks became visible in water and multi‐solvent suppression, and five of these (isopentanol, isobutanol, acetoin, 1,3‐propanediol and myo‐inositol) were included. Only peaks that were not influenced by interleaving artefacts or remaining solvent signals in any of the three methods were considered. The maximum intensity of selected peaks was verified to ensure the correct row extraction. Rows were extracted, and the signal maximum, noise mean and noise standard deviation were calculated using standard Matlab functions. The following equation was used for SNR calculations: $S\mathrm{NR}=\frac{\max_{\text{peak}}}{{\mathrm{std}}_{\text{noise}}}$. A table with precisely selected peaks, extracted rows and noise areas is provided in the Supporting Information (Table S1).

#### 3.2.6 Technical Repeatability

Selected metabolite peaks from SNR calculations were integrated from iuf‐COSY‐msup experiments using a home‐written Matlab script to assess repeatability of the method. Afterwards, the mean, standard deviation and coefficient of variation were calculated from the relative peak volumes. Integration areas, mean relative peak volumes and standard deviation can be found in the Supporting Information (Table S2).

## 4 Results and Discussion

### 4.1 General iuf‐COSY Acquisition and Metabolite Coverage

The interleaved ultrafast COSY with multi‐solvent suppression (iuf‐COSY‐msup) pulse sequence is shown in Figure 1. The suppression block contains a shaped pulse, which selectively excites the previously determined three solvent frequencies of water and ethanol, as well as a NOESY block, to improve faraway water suppression (water nuclei at the edge of the radiofrequency coil). Two additional defocusing gradients (G_1_ and G_2_) further aid solvent suppression. The original approach applied eight individual bands including one for water and seven for each of the peaks of the ethanol triplet and quartet and has been proposed for 1D ^1^H NMR by Monakhova and coworkers to analyse alcoholic beverages, with further adaptation to specific alcoholic beverages, that is, wine or whisky [2, 20, 29]. In the pulse sequence shown in Figure 1, the shaped pulse was placed in the second channel to be independent of the carrier frequency chosen for the iuf‐COSY spectra. This has the advantage that the shaped pulse offset does not need to be recalculated when changing the spectral window position through the carrier frequency in the first channel of the iuf‐COSY spectrum. As described above, an interleaving strategy was used to optimise the compromise between spectral width and resolution, a typical feature of ultrafast experiments [30]. Interleaved scans were recorded with an increasing delay before the bipolar gradient acquisition block, according to $d_{i}=\frac{2\times T_{a}}{N}$ with $d_{i}$ the interleaving delay, $T_{a}$ the duration of the acquisition gradient and N the number of interleaved scans. The spectral width improvement through an interleaved strategy can, however, introduce artefacts that appear systematically at periodic intervals in the conventional dimension. These artefacts have been described in literature and can arise from partial saturation, and also from slight phase, amplitude and time variations in the pulse sequence [13, 30]. They are particularly intense when they arise from strong solvent peaks. In this study, interleaved acquisition enabled the recording of a COSY spectrum spanning the entire aliphatic window (0–6 ppm) on a 700 MHz spectrometer. Accessing a larger spectral width at this magnetic field with the same number of scans would have exceeded an experiment duration of 30 min, which was avoided to prevent damage to the gradient coils. However, at a lower magnetic field and with the same interleaving strategy, the spectral window would be increased [16].

**FIGURE 2:**
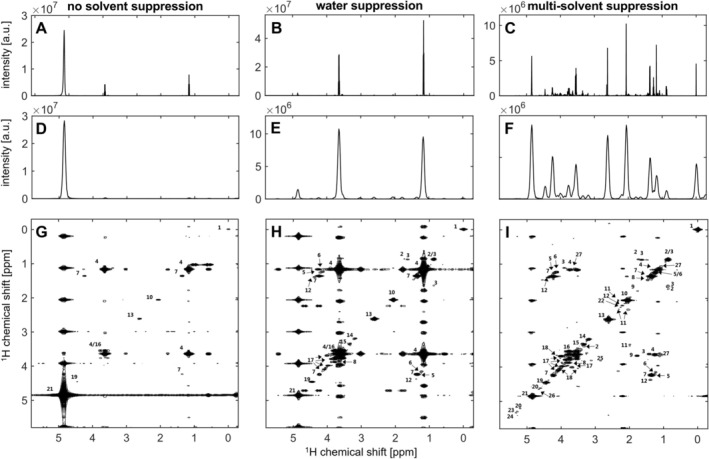
Comparison of suppression schemes: iuf‐COSY spectra without solvent suppression (A, D, G), with water suppression (B, E, H) and multi‐solvent suppression (C, F, I) on a white wine sample recorded on a 700 MHz spectrometer with a cryogenic probe at 298 K. First row shows 1D proton spectra (A–C), second row shows projection of iuf‐COSY in the ultrafast dimension (D–F), third row shows iuf‐COSY spectra (G–I). Annotation: 1: TSP, 2: isobutanol, 3: isopentanol, 4: ethanol, 5: ethyl acetate, 6: ethyl lactate, 7: lactate, 8: alanine, 9: 1,3‐propanediol, 10: acetate, 11: proline, 12: acetoin, 13: succinate, 14: choline, 15: methanol, 16: glycerol, 17: fructose, 18: myo‐inositol, 19: tartrate, 20: glucose, 21: H_2_O, 22: γ‐aminobutyric acid, 23: galacturonic acid, 24: caftaric acid, 25: phenyl ethanol, 26: sugar*, 27: 2,3‐butanediol, 28: ethanolamine (Level 2 annotation, except for *‐labelled, which are Level 3 annotation acc. to [28]).

The iuf‐COSY pulse sequence with multi‐solvent suppression was tested on a white wine sample and compared to experiments with no solvent suppression and with water suppression. The results are shown in Figure 2, which shows the spectra without solvent suppression (Figure 2A,D,G), with water suppression (Figure 2B,E,H) and with multi‐solvent suppression (Figure 2C,F,I). For better comparison, the 1D ^1^H spectra (Figure 2A–C) are shown as well as the projection of the ultrafast dimension (Figure 2D–F) and the iuf‐COSY spectra (Figure 2G–I).

Water (at 4.8 ppm) was the overpowering signal when no solvent suppression was applied (Figure 2G). Only ethanol and a few of the more intense metabolites, such as acetate, lactate, succinate, tartrate and TSP, were observed, as well as systematic interleaving artefacts from the strong solvent peaks. These artefacts can be recognised as ghost peaks along the vertical dimension, with a periodicity that depends on the number of interleaved scans. Depending on the solvent's chemical shift, the overlap with real NMR peaks can become more challenging (Figure 2H). With the suppression of the water signal (Figure 2B,E,H), the receiver gain could be adjusted from 3.56 to 128, with ethanol being the new limiting metabolite intensity for further receiver gain adaptations. A total of 15 metabolites could be detected. With the multi‐solvent suppression technique, the signals from water and ethanol were efficiently suppressed, leading to a final receiver gain adjustment of 724. Especially, the suppression of ethanol cross peaks allowed the observation of other metabolite cross peaks, resulting in a total of 28 annotated metabolites (Figure 2). In addition to optimising the receiver gain, the efficient solvent suppression of water and ethanol signals distinctly reduced the occurrence of systematic interleaving artefacts that overlap with peaks of interest.

A recognised limitation of ultrafast experiments is the sensitivity, which is especially important for metabolomic research [11]. Sensitivity has been optimised in this study through signal averaging and a compromise between sensitivity and spectral window size. However, ultrafast experiments are devoid of t_1_ noise, which is especially interesting in wine, where distinct concentration differences are possible (e.g., organic acids, sugars and phenols). Additionally, the advantage of 2D correlation experiments lies in the cross peaks, which provide additional structural information and a lower degree of signal overlap compared to their diagonal counterparts. Both factors enable better annotation, integration and optional quantification when combined with a calibration method [31]. In this specific wine sample, the cross peaks of 13 metabolites were observed and annotated (Figure 2I). These include isobutanol, isopentanol, 2,3‐butanediol, 1,3‐propanediol, proline, ethyl acetate, ethyl lactate, lactate, acetoin, glycerol, ethanolamine, myo‐inositol and fructose. Some of these metabolites have been reported to be crucial for the flavour of wines, while others enable differentiation of grape variety or geographical location.

Overall, many of the metabolites annotated in this sample using iuf‐COSY‐msup were reported to be relevant for differentiating wines according to various locations, terroirs, varieties, and vintages. Examples included Cabernet Sauvignon from France, the USA, and Australia, La Rioja wines from various terroirs in Spain, different white wine varieties in Germany, and several vintages of Bordeaux wines in France [2, 3, 5, 32].

### 4.2 Signal‐to‐Noise Ratio Calculation

**FIGURE 3:**
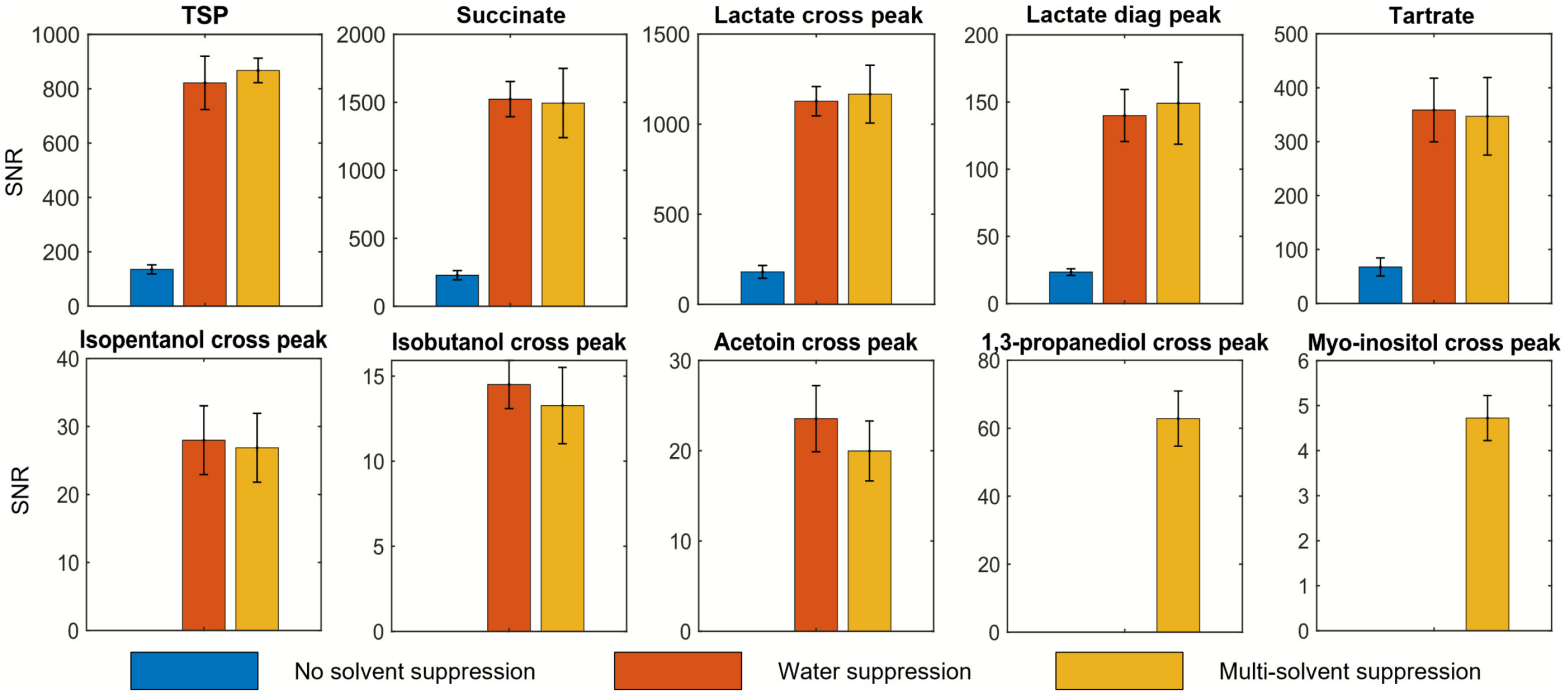
Signal‐to‐noise ratio (SNR) comparison of 10 metabolite peaks from iuf‐COSY spectra between no solvent (blue), water (orange) and multi‐solvent suppression methods (yellow). Bars represent mean SNR with error bars for standard deviation (*n* = 5), missing bars indicate undetected peaks.

An analytical parameter to evaluate the new suppression scheme, apart from the improved number of metabolite observations, is the comparison of the signal‐to‐noise ratio (SNR) of established methods and the new one. However, only five metabolites (TSP, lactate, acetate, succinate and tartrate) are high enough in concentration to be observable in all three methods. Furthermore, only peaks unaffected by strong solvent signals or interleaving artefacts in any of the three methods were chosen for SNR calculation, with off‐diagonal peaks preferred for analysis where possible. Lactate was the only metabolite to show a cross peak signal in the spectrum without solvent suppression and was therefore measured on‐ and off‐diagonal. Isopentanol, isobutanol and acetoin cross peaks were chosen as an example for metabolites appearing in water and multi‐solvent suppression, while 1,3‐propanediol and myo‐inositol cross peaks are examples of metabolites only observed in multi‐solvent suppression. The calculated results from five repetitions per method are shown in Figure 3.

In general, SNR would be expected to increase with improved suppression of solvent signals, due to the adaptation of the instrument receiver gain from 3.56 for no solvent suppression to 128 for water suppression and a final optimisation of 724 for multi‐solvent suppression. Figure 3 shows a distinct increase in SNR from no solvent suppression to either water or multi‐solvent suppression for TSP, succinate, lactate and tartrate. The ratio of signal‐to‐noise between water and multi‐solvent suppression was of the same magnitude for metabolite peaks that were detected in both methods. A possible explanation could be that an SNR plateau has been reached, as observed by Peters and coworkers in their work [33]. They tested different levels of receiver gain on different hyperpolarised nuclei and different NMR spectrometers (1, 7, 9.4, 11.7 and 14.1 T) and evaluated the signal intensity and SNR. From their findings, they proposed that an increasing receiver gain linearly increased the signal intensity while the SNR levelled out into an asymptotic plateau after an initial improvement. This compares with what has been observed with this wine sample, where the signal intensity increased linearly with the increased receiver gain between the methods (see Figure S5), while the SNR levelled off in water and multi‐solvent suppression.

Despite this SNR plateau, the suppression of ethanol signals and the reduction of interleaving artefacts enabled the detection of 11 more metabolites compared to a total of 15 compounds with water suppression alone. 1,3‐propanediol and myo‐inositol are depicted in Figure 3 as examples of cross peaks that can only be accessed in the case of multi‐solvent suppression.

### 4.3 Repeatability of Relative Peak Volumes

Repeatability is a key factor in metabolomic studies and authentication analysis. This was therefore evaluated using the same 10 metabolite signals as in the SNR analysis, by calculating the coefficient of variation (CV) on 2D peak volumes across five replicates (Table 1). The variation for signals was determined to be between 0.2% and 6.1%. More concentrated metabolites, such as succinate, lactate and tartrate, showed a small coefficient of variation of less than 1%. Signals with an SNR lower than 100 showed slightly higher variation (1.5%–6.1%).

**TABLE 1:** Repeatability of relative peak volumes for iuf‐COSY‐msup (*n* = 5), measured on 10 metabolites shown as coefficient of variation (CV) in %.

Metabolite	CV (%)
TSP	0.41
Succinate	0.32
Lactate cross peak	0.18
Lactate diagonal peak	0.96
Tartrate	0.56
Isopentanol cross peak	3.15
Isobutanol cross peak	1.49
Acetoin cross peak	5.61
1,3‐Propanediol cross peak	2.31
Myo‐inositol cross peak	6.06

This is in the same range as the technical repeatability reported in a previous study of ultrafast techniques on tomato fruit extract, where the variation was between 1.0% and 6.4% on the same magnetic field strength [16]. This highlights the characteristic high repeatability of iuf‐COSY, which is less sensitive to spectrometer instabilities and has no t_1_ noise compared to conventional 2D methods [14, 15]. In comparison, an interlaboratory ring test evaluated the possibility of the 1D ^1^H multi‐solvent suppression method for wine authentication in an international collaboration [34]. They reported intra‐laboratory repeatability based on absolute quantification of 1.0%–11.0% for glucose, 1.4%–4.6% for acetic acid, 1.7%–23.0% for malic acid and 1.3%–71.5% for fumaric acid, among others. In contrast, the here presented method evaluated different metabolites and focused on technical repeatability based on relative peak volumes, yet the results still demonstrate acceptable repeatability levels.

### 4.4 Artefact Removal Through Optimised Coherence Selection

**FIGURE 4:**
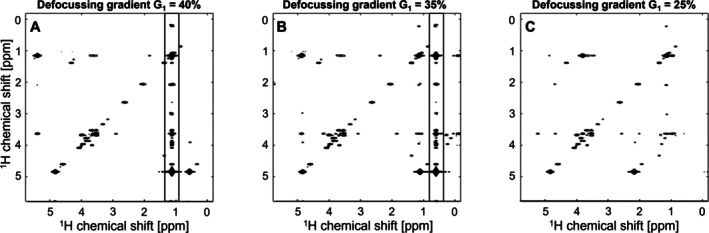
Impact of adaptation of defocusing gradient G_1_ on vertical artefacts (A–C) observed during optimisation process of iuf‐COSY with multi‐solvent suppression. Gradient percentages translate to (A) G_1_ = 24.9 G/cm, (B) G_1_ = 21.8 G/cm and (C) G_1_ = 15.6 G/cm.

During the optimisation process of the iuf‐COSY‐msup, vertical artefact lines in the conventional dimension were observed, which initially limited the ability to adapt the receiver gain (Figure 4A,B). This is likely because the additional defocusing gradient from the solvent suppression block compensates coherence selection gradients when they are set to their default values. By adapting the defocusing gradient G_1_, the artefacts could be moved outside the spectral window (Figure 4C), which led to a further adaptation of the receiver gain. The results presented above are based on these optimised parameters.

### 4.5 Comparison of iuf‐COSY With Multi‐Solvent Versus WET Suppression

**FIGURE 5:**
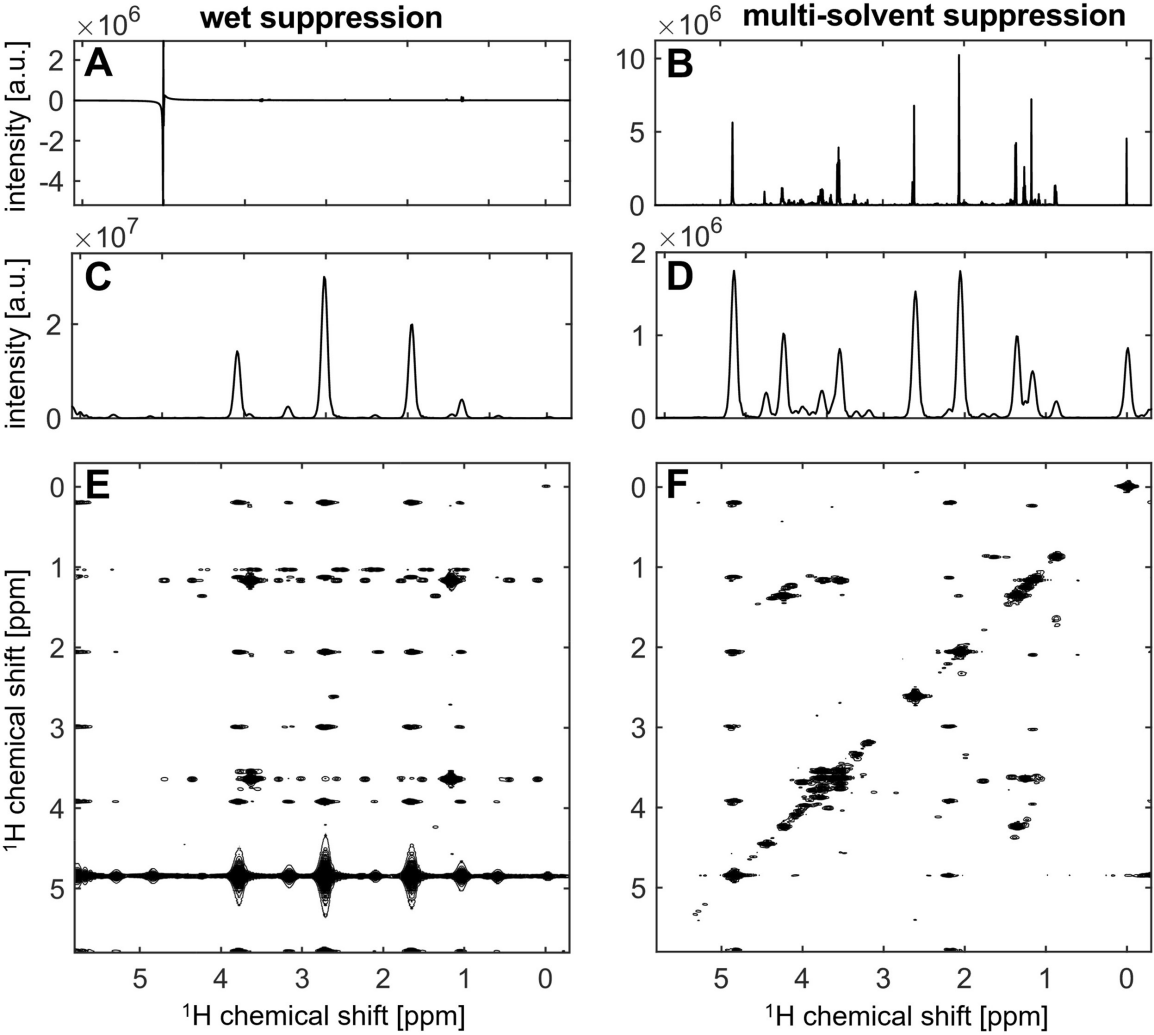
Comparison of methods: iuf‐COSY spectra with WET (A, C, E) and multi‐solvent suppression (B, D, F) on a white wine sample of Muscadet recorded on a 700 MHz spectrometer at 298 K. First row shows 1D proton spectra (A and B), second row shows projection of iuf‐COSY in the ultra‐fast dimension (C and D) and third row shows iuf‐COSY spectra (E and F).

iuf‐COSY has been previously combined with WET (iuf‐COSY‐WET) to suppress methanol in a reaction monitoring experiment using flow NMR [35]. Since WET can be optimised to suppress several solvent signals, this suppression scheme was tested on the wine sample. The comparison between iuf‐COSY‐WET (Figure 5A,C,E) and multi‐solvent suppression (Figure 5B,D,F) can be observed in Figure 5, with Figure 5A,B showing 1D ^1^H spectra, Figure 5C,D presenting the projection of the ultrafast dimension and Figure 5E,F picturing the iuf‐COSY spectra.

From Figure 5A, which shows the proton spectra using WET suppression, it is evident that while the ethanol signals appear to be well suppressed, this was not the case for the water signal despite extensive optimisation tests. The unsuppressed solvent signals were likely to be the reason for the strong systematic interleaving artefacts observed in the iuf‐COSY‐WET. In comparison, interleaving artefacts were already visible in Figure 2 between the iuf‐COSY spectra without water (Figure 2G), with water (Figure 2H) and with multi‐solvent suppression (Figure 2I), although to a lesser extent.

Overall, the iuf‐COSY‐WET did not provide suppression results comparable to those of the presaturation method and was strongly dominated by systematic interleaving artefacts. Lhoste and coworkers experienced similar artefacts when suppressing either MeOH or CHCl_3_ as solvents in their work using iuf‐COSY‐WET in a flow set‐up [35]. However, they observed a distinct spectral improvement when changing their encoding axis from z‐ to x‐axis with a triple‐axis gradient broadband inversion‐detection probe. It is likely that they experienced these interleaving artefacts due to the flow direction of the flow tube in the z‐axis direction, which does not apply to the static wine sample used in this experiment.

### 4.6 High‐Throughput and Other Applications

The iuf‐COSY‐msup spectrum shown in this study was recorded in 23 min. This duration was chosen intentionally, as a compromise between throughput and sensitivity, and to preserve the integrity of the gradient coil. This is comparable to the timescale of 1D ^1^H acquisitions used in wine profiling studies [4, 36]. However, this compromise of throughput and sensitivity can be adapted to the specific research question, making the iuf‐COSY method an interesting candidate approach for future high‐throughput wine profiling studies. It is, however, important to underline that iuf‐COSY, together with other fast 2D NMR methods, should be seen as a complement to 1D NMR analysis. As shown in recent metabolomics studies, 2D NMR generally performs better to highlight more concentrated biomarkers that are overlapped in 1D NMR, while 1D NMR remains more sensitive to detect low‐concentration biomarkers [17, 18]. The general power of the iuf‐COSY method lies in the possibility of recording a 2D spectrum within one single scan. In the proposed method, a longer experiment time is required to achieve a sufficient spectral width and sensitivity, enabling phase cycling. Depending on the application and the sample analysed, it might be interesting to acquire single‐scan spectra, for example, for flow monitoring or samples of highly concentrated extractions. To serve these needs, an adapted version of the iuf‐COSY with multi‐solvent suppression consisting of only the shaped pulse and one defocusing gradient is provided (pulse sequence Figure S6). Tests on the wine sample (Figure S7) showed well‐suppressed ethanol signals, but less effective water suppression, which resulted in more interleaving artefacts. However, depending on the research question, different options have been proposed, and researchers can consider which method fits their needs better.

The results presented in this paper should also be discussed in light of the previous studies that employed ultrafast 2D NMR (and more specifically the iuf‐COSY pulse sequence) for the study of complex mixtures. The comparison between ultrafast 2D COSY and its conventional counterpart has been reported in detail [14, 15]. Briefly, due to limitations that are inherent to the method, the sensitivity for iuf‐COSY is 5–10 times less than that of a 1D spectrum with the same acquisition time, and the iuf‐COSY method also includes a trade‐off between resolution and spectral widths [11]. However, the main advantage of iuf‐COSY lies in the absence of t_1_ noise compared to conventional COSY [14]. While this advantage can be partially reduced in favourable conditions (e.g., fast conventional COSY with a short recovery delay), it results in a better repeatability of iuf‐COSY. In practice, this is true for experiment times below 30 min. When low analyte concentrations require more than 30 min of signal averaging, ultrafast 2D NMR methods are not recommended to preserve the integrity of the gradient coil. As a consequence, iuf‐COSY experiments should be seen as a part of a toolbox for complex mixture analysis, which includes a range of fast 2D NMR experiments with complementary time‐saving methods [37].

## 5 Conclusion

The proposed iuf‐COSY method demonstrates an efficient multi‐solvent suppression approach for high‐throughput analysis of wine samples. The iuf‐COSY spectrum of a wine sample can be recorded in approximately 20 min, providing reasonable high‐throughput capabilities, enhanced structural information through correlation peaks and improved peak distribution compared to crowded regions in standard 1D ^1^H experiments. The iuf‐COSY experiment is, therefore, an interesting option for future metabolomic and authentication studies aiming to apply 2D NMR methods for high‐throughput wine analysis.

## Conflicts of Interest

Patrick Giraudeau is the Deputy Editor of Magnetic Resonance in Chemistry. In accordance with the journal's policy and COPE guidelines, the manuscript was handled by an independent editor to ensure a fair and unbiased review process.

## Supporting information


**Figure S1:** 1D ^1^H pulse sequence with multi‐solvent suppression scheme.
**Figure S2:** Pulse sequence of interleaved ultrafast COSY with continuous‐wave presaturation suppression (iuf‐COSY‐pr).
**Figure S3:** Pulse sequence of interleaved ultrafast COSY with WET (iuf‐COSY‐wet)
**Figure S4:** TOCSY NUS for metabolite identification with multi‐solvent suppression
**Figure S5:** Correlation plots of maximum signal intensity and receiver gain (RG)
**Figure S6:** Single‐scan version (no phase cycling) of the ultrafast COSY with multi‐solvent suppression.
**Figure S7:** Comparison of iuf‐COSY spectra with multi‐solvent suppression
**Table S1:** Extra information for signal‐to‐noise ratio calculations.
**Table S2:** Extra information for repeatability measurements.

## Data Availability

The data supporting the findings of this study are available on the Zenodo repository [38].
