# Supplementary material for: Multi‐Solvent Suppression Ultrafast 2D COSY for High‐Throughput Wine Screening
Source: Magn Reson Chem. 2026 Jan 18;64(4):416–26. doi: 10.1002/mrc.70078 (PMC12950335; doi:10.1002/mrc.70078)
Supplement: Supplementary file 1 — Figure S1: 1D 1H pulse sequence with multi‐solvent suppression scheme. Figure S2: Pulse sequence of interleaved ultrafast COSY with continuous‐wave presaturation suppression (iuf‐COSY‐pr). Figure S3: Pulse sequence of interleaved ultrafast COSY with WET (iuf‐COSY‐wet) Figure S4: TOCSY NUS for metabolite identification with multi‐solvent suppression Figure S5: Correlation plots of maximum signal intensity and receiver gain (RG) Figure S6: Single‐scan version (no phase cycling) of the ultrafast COSY with multi‐solvent suppression. Figure S7: Comparison of iuf‐COSY spectra with multi‐solvent suppression Table S1: Extra information for signal‐to‐noise ratio calculations. Table S2: Extra information for repeatability measurements. [file MRC-64-416-s001.docx]

**Supplemental Material:**

**Multi-solvent suppression ultrafast 2D COSY for high-throughput wine screening**

Pia S. Mayer^1^*, Jérémy Marchand^2^, Marine P.M. Letertre^2^, Jean-Nicolas Dumez^2^, Søren B. Engelsen^1^ and Patrick Giraudeau^2^*

^1^ Department of Food Science, University of Copenhagen, Rolighedsvej 26, 1958, Frederiksberg C, Denmark

^2^ Nantes Université, CNRS, CEISAM, UMR 6230, Nantes, France

*correspondence to: pia.mayer@food.ku.dk and patrick.giraudeau@univ-nantes.fr

**Table of Contents**

[1. **Tables with details for analytical calculations** 2](#_Toc210146090)

[**Table ST1:** Extra information for signal-to-noise ratio calculations. 2](#_Toc210146091)

[**Table ST2:** Extra information for repeatability measurements. 3](#_Toc210146092)

[2. **Additional pulse sequences** 3](#_Toc210146093)

[**Figure S1:** 1D ^1^H pulse sequence with multi-solvent suppression scheme. 3](#_Toc210146094)

[**Figure S2:** Pulse sequence of interleaved ultrafast COSY with continuous-wave presaturation suppression (iuf-COSY-pr). 4](#_Toc210146095)

[**Figure S3:** Pulse sequence of interleaved ultrafast COSY with WET (iuf-COSY-wet). 4](#_Toc210146096)

[3. **Additional information on metabolite identification from TOCSY** 5](#_Toc210146097)

[**Figure S4:** TOCSY NUS for metabolite identification with multi-solvent suppression 5](#_Toc210146098)

[4. **Signal intensity vs receiver gain** 6](#_Toc210146099)

[**Figure S5:** Correlation plots of maximum signal intensity and receiver gain (RG) 6](#_Toc210146100)

[5. **Adaptation of iuf-COSY-msup for single-scan: pulse sequence and spectrum** 6](#_Toc210146101)

[**Figure S6:** Single-scan version (no phase cycling) of the ultrafast COSY with multi-solvent suppression. 6](#_Toc210146102)

[**Figure S7:** Comparison of iuf-COSY spectra with multi-solvent suppression 7](#_Toc210146103)

[6. **Reference** 7](#_Toc210146104)

# Tables with details for analytical calculations

## **Table ST1:** Extra information for signal-to-noise ratio calculations.

The table displays the respective signal and noise areas, as well as the exact rows that has been extracted for calculations.

|  |  | **TSP** | **Succinate** | **Lactate cross peak** | **Lactate diagonal peak** | **Tartrate** | **Isopentanol cross peak** | **Isobutanol cross peak** | **Acetoin cross peak** | **1,3-propanediol cross peak** | **Myo-inositol cross peak** |
| --- | --- | --- | --- | --- | --- | --- | --- | --- | --- | --- | --- |
| **signal area** | start [ppm] | -0.3 | 2.33 | 4 | 4 | 4.2 | 0.7 | 0.7 | 1.24 | 1.6 | 3.1 |
|  | end [ppm] | 0.3 | 2.8 | 4.5 | 4.5 | 4.6 | 1 | 1 | 1.6 | 1.9 | 3.4 |
| **noise area** | start [ppm] | 2 | 0 | 2.2 | 2.2 | 0 | -0.1 | 1.35 | -0.5 | -0.4 | -0.5 |
|  | end [ppm] | 3 | 1 | 3.2 | 3.2 | 1 | 0.5 | 2.35 | 0.5 | 0.6 | 0.5 |
| **no suppression** | row rep. 1 | 918 | 558 | 730 | 335 | 305 | n.d. | n.d. | n.d. | n.d. | n.d. |
|  | row rep. 2 | 918 | 558 | 730 | 334 | 305 | n.d. | n.d. | n.d. | n.d. | n.d. |
|  | row rep. 3 | 918 | 558 | 730 | 335 | 305 | n.d. | n.d. | n.d. | n.d. | n.d. |
|  | row rep. 4 | 918 | 558 | 730 | 335 | 305 | n.d. | n.d. | n.d. | n.d. | n.d. |
|  | row rep. 5 | 918 | 558 | 730 | 334 | 305 | n.d. | n.d. | n.d. | n.d. | n.d. |
| **water suppression** | row rep. 1 | 918 | 558 | 730 | 334 | 305 | 692 | 680 | 316 | n.d. | n.d. |
|  | row rep. 2 | 918 | 558 | 730 | 334 | 305 | 691 | 681 | 316 | n.d. | n.d. |
|  | row rep. 3 | 918 | 558 | 730 | 335 | 305 | 692 | 680 | 316 | n.d. | n.d. |
|  | row rep. 4 | 918 | 558 | 730 | 334 | 305 | 692 | 680 | 316 | n.d. | n.d. |
|  | row rep. 5 | 918 | 558 | 730 | 334 | 305 | 691 | 682 | 316 | n.d. | n.d. |
| **multi-solvent suppression** | row rep. 1 | 918 | 558 | 730 | 335 | 305 | 691 | 681 | 315 | 412 | 423 |
|  | row rep. 2 | 918 | 558 | 730 | 335 | 305 | 691 | 679 | 315 | 412 | 423 |
|  | row rep. 3 | 918 | 558 | 730 | 335 | 305 | 692 | 680 | 315 | 412 | 423 |
|  | row rep. 4 | 918 | 558 | 730 | 335 | 305 | 692 | 680 | 316 | 412 | 423 |
|  | row rep. 5 | 918 | 558 | 730 | 335 | 305 | 691 | 679 | 316 | 412 | 423 |

rep: repetition; n.d.: not detected

## **Table ST2:** Extra information for repeatability measurements.

Technical repeatability measurement of iuf-COSY-msup from five replicates on 10 selected metabolite peaks. The table provides the chemical shifts of integration areas, means, and standard deviations of the relative peak volumes used in the following calculations.

| **Metabolite** | **F2 start [ppm]** | **F2 end [ppm]** | **F1 start [start]** | **F1 end [ppm]** | **Mean [rel.vol.]** | **Standard Deviation [rel. vol.]** |
| --- | --- | --- | --- | --- | --- | --- |
| TSP | -0.20 | 0.19 | -0.10 | 0.08 | 21541803 | 88276 |
| Succinate | 2.35 | 2.84 | 2.52 | 2.73 | 43921464 | 142625 |
| Lactate cross peak | 4.01 | 4.47 | 1.29 | 1.42 | 28853529 | 51765 |
| Lactate diagonal peak | 4.10 | 4.35 | 4.18 | 4.30 | 5378347 | 51671 |
| Tartrate | 4.30 | 4.58 | 4.39 | 4.52 | 8236051 | 46516 |
| Isopentanol cross peak | 0.78 | 0.96 | 1.58 | 1.68 | 1245621 | 39198 |
| Isobutanol cross peak | 0.79 | 0.94 | 1.69 | 1.76 | 552065 | 8222 |
| Acetoin cross peak | 1.25 | 1.52 | 4.32 | 4.42 | 1165312 | 65361 |
| 1,3-propanediol cross peak | 1.66 | 1.88 | 3.62 | 3.72 | 2067409 | 47688 |
| Myo-inositol cross peak | 3.13 | 3.35 | 3.56 | 3.61 | 429524 | 26020 |

# Additional pulse sequences


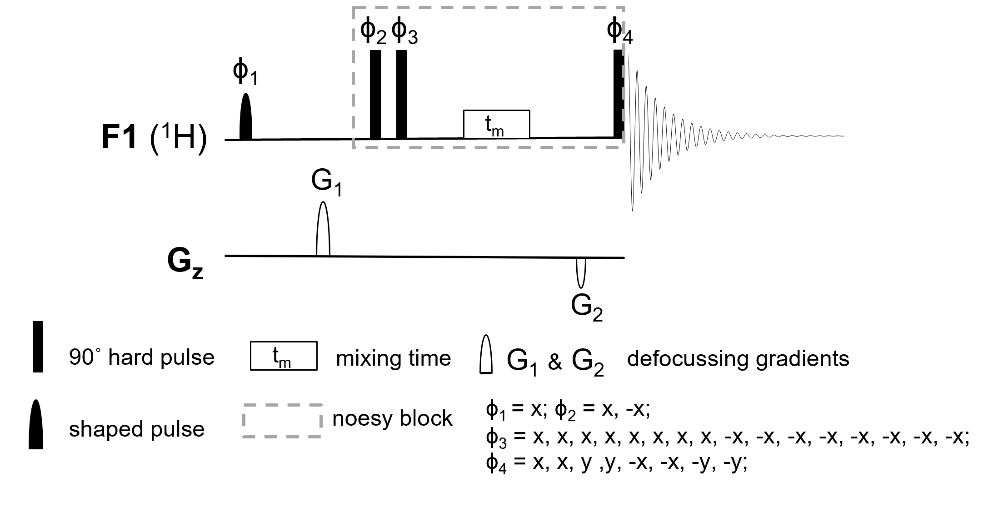


## **Figure S1:** 1D ^1^H pulse sequence with multi-solvent suppression scheme.


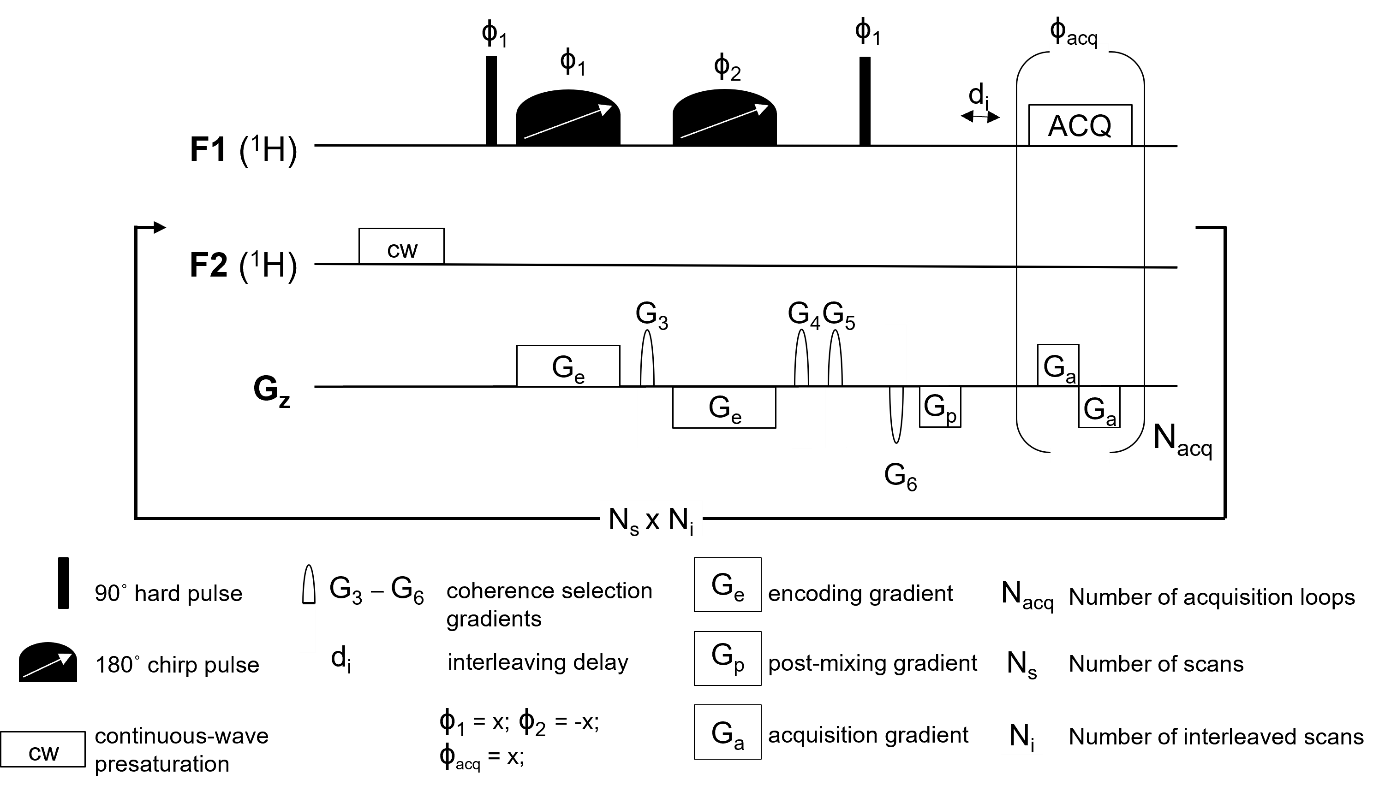


## **Figure S2:** Pulse sequence of interleaved ultrafast COSY with continuous-wave presaturation suppression (iuf-COSY-pr).


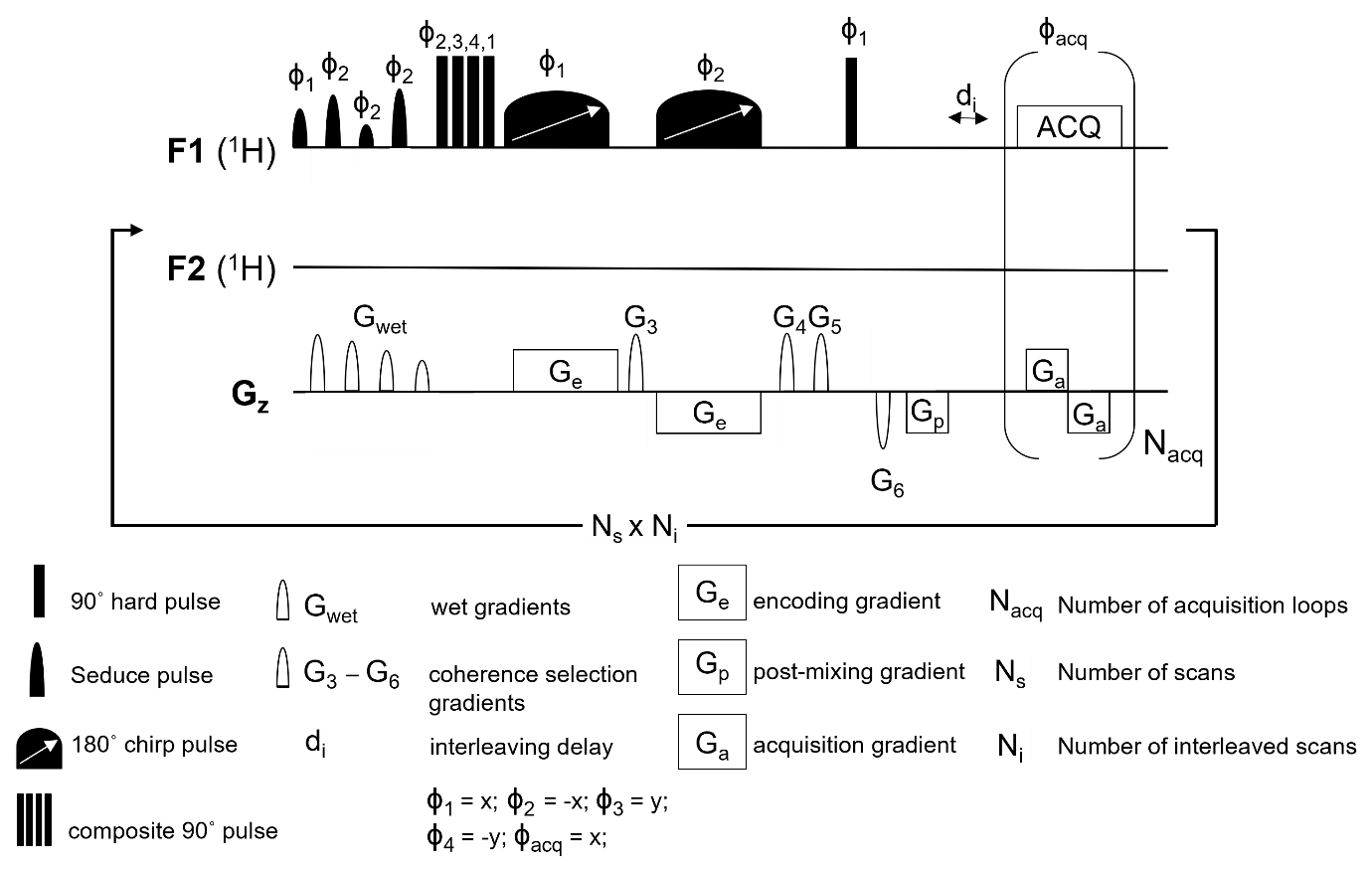


## **Figure S3:** Pulse sequence of interleaved ultrafast COSY with WET (iuf-COSY-wet).

# Additional information on metabolite identification from TOCSY


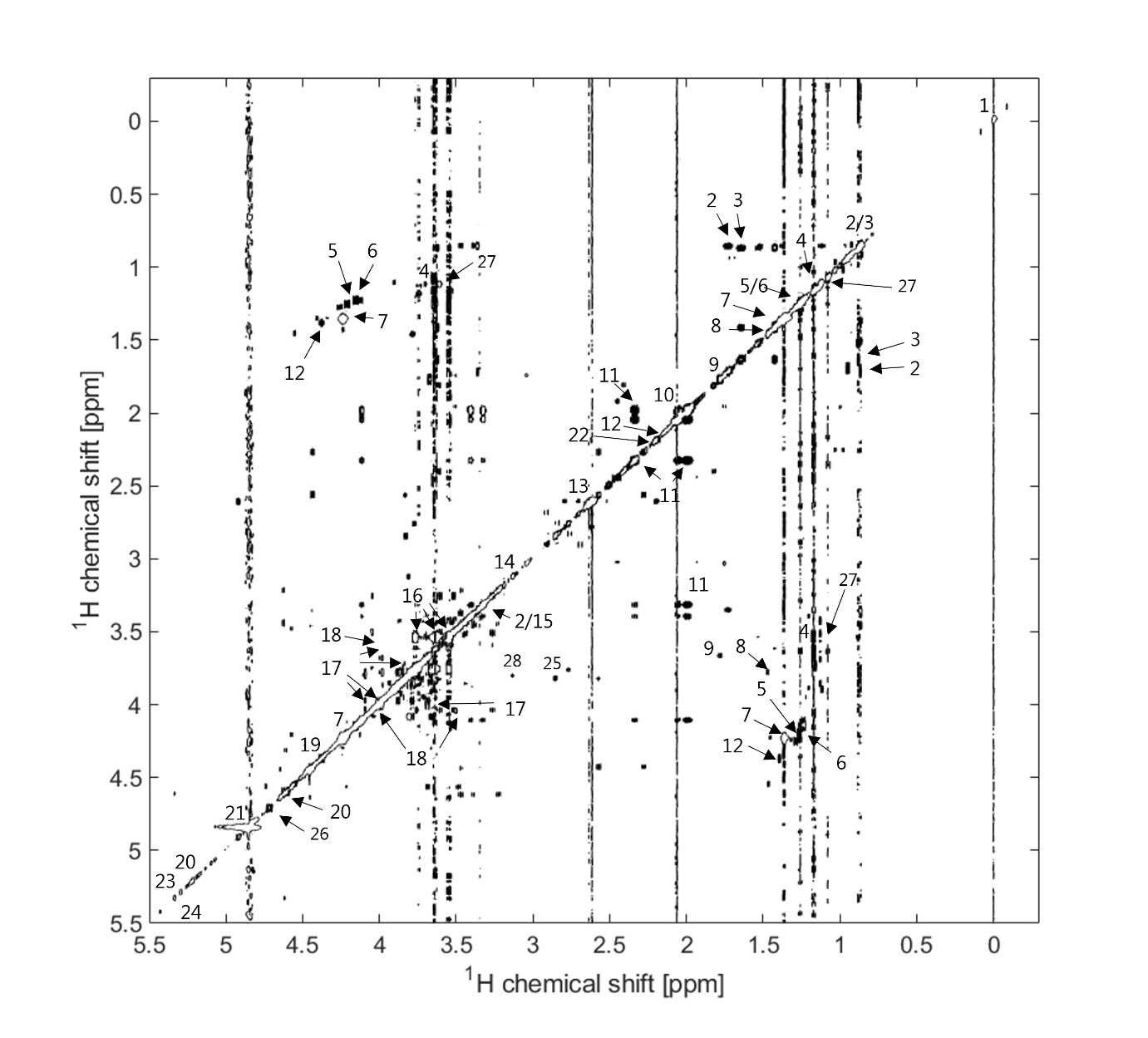


## **Figure S4:** TOCSY NUS for metabolite identification with multi-solvent suppression

on a white wine sample recorded on a 700 MHz spectrometer at 298 K. Annotation: 1: TSP, 2: isobutanol, 3: isopentanol, 4: ethanol, 5: ethyl acetate, 6: ethyl lactate, 7: lactate, 8: alanine, 9: 1,3-propanediol, 10: acetate, 11: proline, 12: acetoin, 13: succinate, 14: choline, 15: methanol, 16: glycerol, 17: fructose, 18: myo-inositol, 19: tartrate, 20: glucose, 21: H_2_O, 22: γ­-aminobutyric acid, 23: galacturonic acid, 24: caftaric acid, 25: phenyl ethanol, 26: sugar*, 27: 2,3-butanediol, 28: ethanolamine (Level 2 annotation, except for *-labelled, which are Level 3 annotation acc. to [1]).

# Signal intensity vs receiver gain


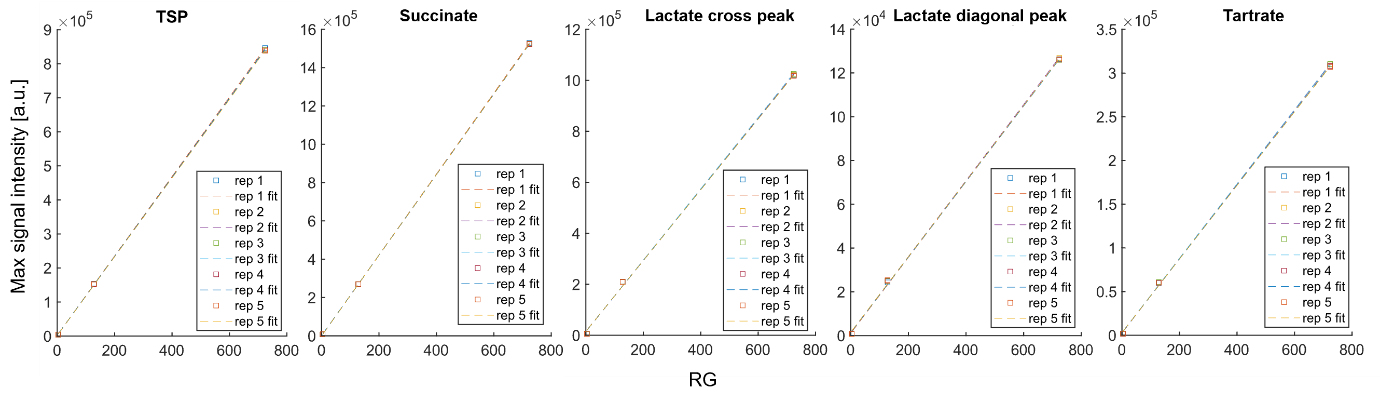


## **Figure S5:** Correlation plots of maximum signal intensity and receiver gain (RG)

of TSP, succinate, lactate cross peak, lactate diagonal peak and tartrate (peaks that showed up in all three methods). RG was optimised for each method: RG = 3.56 for no solvent suppression, RG = 128 for water suppression, and RG = 724 for and multi-solvent suppression. Each method was acquired with five replicates.

# Adaptation of iuf-COSY-msup for single-scan: pulse sequence and spectrum


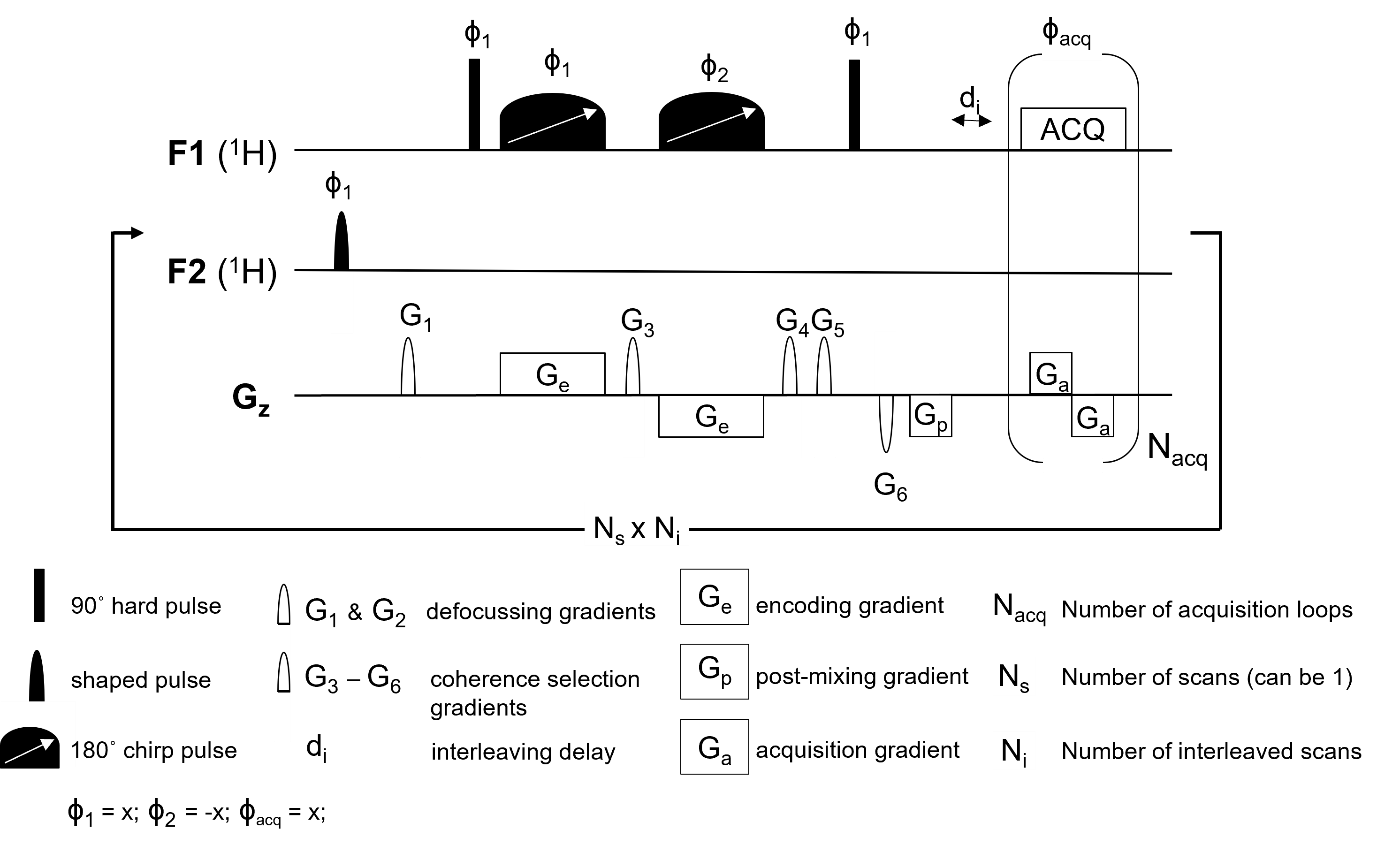


## **Figure S6:** Single-scan version (no phase cycling) of the ultrafast COSY with multi-solvent suppression.


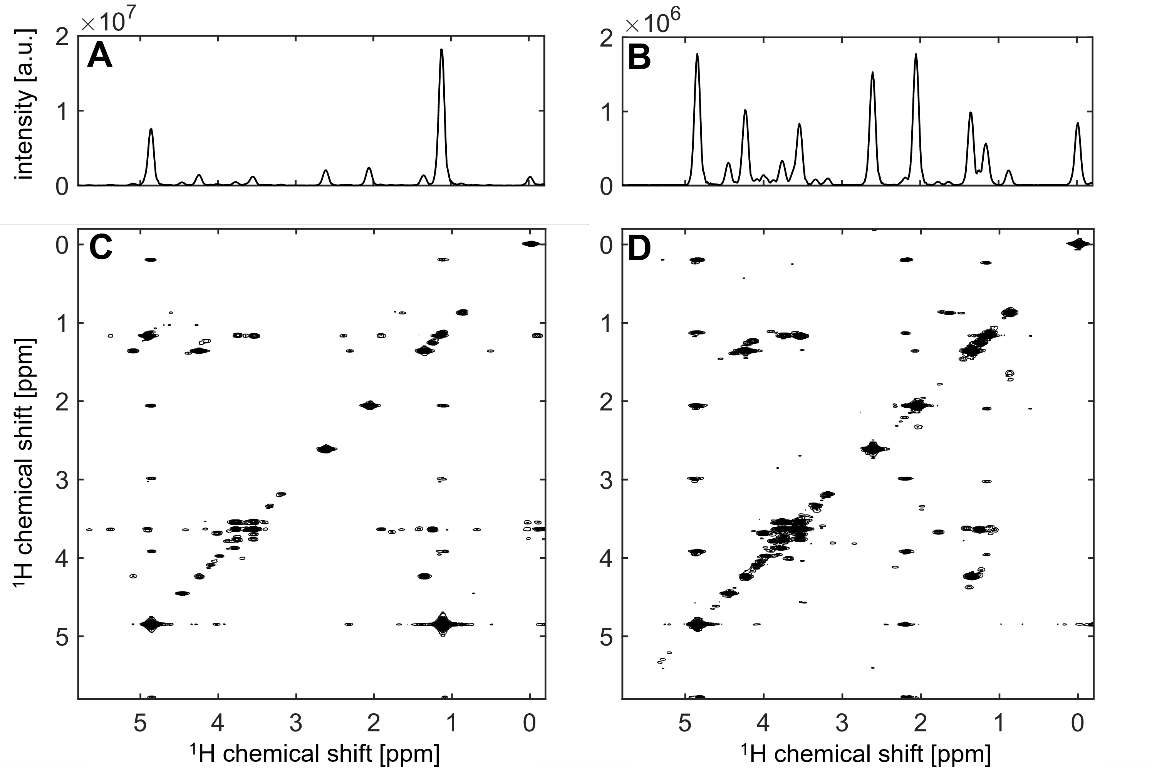


## **Figure S7:** Comparison of iuf-COSY spectra with multi-solvent suppression

of a white wine sample recorded on a 700 MHz spectrometer at 298 K. Results of suppression with shaped pulse and one defocusing gradient (pulse sequence Figure S4) in A & C, while iuf-COSY-msup (pulse sequence Figure 1) is shown in B & D. A & B show projections of iuf-COSY in the ultra-fast dimension. C & D show respective iuf-COSY spectra.

# Reference

[1] L. W. Sumner, A. Amberg, D. Barrett *et al.*, "Proposed minimum reporting standards for chemical analysis," *Metabolomics*  3 (2007): 211-221, doi: 10.1007/s11306-007-0082-2.
